# Predictive and Contemporaneous Power of the Determinants of Stock Liquidity

**DOI:** 10.3389/fpsyg.2022.912159

**Published:** 2022-07-27

**Authors:** Linyin Xie, Yuanqing Jin, Chanxuan Mo

**Affiliations:** ^1^College of Science and Technology, Ningbo University, Ningbo, China; ^2^University of Nottingham Ningbo China, Ningbo, China

**Keywords:** China, idiosyncratic, systematic, stock liquidity determinants, liquidity risk management

## Abstract

This study constructs nine (five) idiosyncratic (systematic) variables to test the predictive and contemporaneous power of the determinants of stock liquidity. We select A shares from January 2003 to September 2021 to study stock liquidity in the Chinese market. As a novel discovery, this study finds that stock liquidity abnormally decreases with contemporaneous idiosyncratic return and also with 1-year lagged systematic return. Only the idiosyncratic return variance can decrease future short-term or contemporaneous stock liquidity. Idiosyncratic factors are more important than systematic ones for contemporaneous and future stock liquidity. The predictive power of the determinants decreases with the forecast length. Economic policy uncertainty (EPU) can affect sensitivity of stock liquidity to contemporaneous determinants. The empirical results of this research are robust over subperiods, forecast length and across four liquidity measurements. The abnormalities and linkages between determinants and stock liquidity are correlated with investor psychology and special market mechanism in China.

## Introduction

Liquidity is an important financial concept. At the microlevel, stock liquidity is closely associated with corporate value. At the macro-level, the 1997 Asian financial crisis and the 2008 American subprime mortgage crisis show that credit default risk and currency depreciation risk can be contagious to the stock market, causing the simultaneous drying up of stock liquidity and further triggering the global financial crisis. Exploring liquidity determinants is a key part of controlling stock liquidity risk and managing asset wealth.

The measurement, determinants, and effects of stock liquidity are the three main topics in this area. As Kyle (1985) deems liquidity a slippery and elusive concept, various liquidity measures exist, and research has been focused on seeking better methods to measure stock liquidity. Exploring liquidity determinants is a better approach toward understanding stock liquidity. The stock price, trading volume, and return volatility of individual stocks are three important and traditional micro-determinants of stock liquidity (Chordia et al., 2001; Rösch and Kaserer, 2013). Institutional ownership is also an important determinant. The latest research specifies institutional ownership into domestic, foreign, long-term, and short-term classifications and studies how these different forms of institutional ownership affect stock liquidity (Ding et al., 2017; Lee and Chung, 2018; Wang and Wei, 2021). Information demand and supply are the new determinants of stock liquidity. They have been studied in the form of investor attention, media coverage, and episodes of sensational news exogenous to the market (Aouadi et al., 2018; Peress and Schmidt, 2020; Shyu et al., 2020; Cheng et al., 2021). Market structure reforms, such as state ownership transformation and tick size pilot programs, are still popular determinants of stock liquidity (Boubakri et al., 2020; Chung et al., 2020). Research has been conducted on the determinants of stock liquidity from very fresh perspectives, such as lawyer CEOs, mandatory CSR expenditure, as well as oil supply and demand shocks (Pham, 2020; Roy et al., 2022; Zhang and Wong, 2022). These determinants affect stock liquidity through the channels of inventory risk, information asymmetry, and funding liquidity. The theoretical linkage between determinants and stock liquidity has mostly been explained from a psychological perspective.

This study makes three novel contributions to the literature. First, it expands the research field on how stock return and volatility change stock liquidity. Most of the previous research has focused on market or individual returns and volatility. This study splits individual returns and variances into predictable and residual parts. They are found to have an abnormal impact on stock liquidity. Second, this study examines the relationship between stock liquidity and uncertainty in an innovative manner. In addition to studying how uncertainty affects stock liquidity, previous research studies the determinants and pricing of the uncertainty elasticity of liquidity (Chung and Chuwonganant, 2014; Rehse et al., 2019; Chiu, 2020; Sun et al., 2021). This study focuses on how uncertainty affects the sensitivity of stock liquidity to determinants. Third, this study enriches the Chinese empirical literature on the determinants of stock liquidity. The latest research on Chinese stock liquidity mostly emphasizes regulatory reforms, such as short selling, margin trading, split-share structure reform, and the Shanghai–Hong Kong stock connect (Li et al., 2018; Ma et al., 2018; Ye et al., 2020). This study examines stock liquidity determinants from the systematic and idiosyncratic aspects, as well as different predictive lengths. As the Chinese market lacks information transparency and investment protection and has a different market mechanism, this study finds results that differ from previous findings in the American or Euro market.

The remainder of this article is organized as follows: Section 2 presents the related literature. Section 3 discusses the data source, sample construction, variable measurement, and descriptive statistics. Sections 4 and 5 investigate the contemporaneous and future impact of systematic and idiosyncratic determinants on stock liquidity. Robustness tests are conducted over four subperiods, different forecast length and across four liquidity proxies.

## Related Literature Review

Inventory risk and information asymmetry are classical theoretical frameworks that determine stock liquidity (Chordia et al., 2001). Amihud et al. (2013) propose a liquidity spiral between both market and funding liquidity. This section mainly theoretically elucidates how stock liquidity is determined through the channels of inventory, information asymmetry, and funding liquidity, and then exhibits empirical findings in the Chinese market.

### Theoretical Effect of Idiosyncratic Factors on Stock Liquidity

Idiosyncratic factors enhance or deteriorate stock liquidity through the channels of inventory or information asymmetry. The impact may be diversified, even through only one channel.

Individual stock return volatility and financial leverage are expected to be negatively associated with stock liquidity through inventory and information asymmetry. Chordia et al. (2001) argue that volatility increases the stock bid–ask spread through the channel of inventory risk and trading activity through the risk of engaging in short-term speculative activity. Inventory risk increases with stock return volatility. The bid–ask spread is widened to compensate for inventory risk, and trading depth is increased to avoid costs caused by inventory burden. Additionally, Benston and Hagerman (1974) argue that individual stock volatility has a cross-sectional association with a higher bid–ask that spreads through the channel of asymmetric information. For financial leverage, static trade-off theory posits that a firm's capital-raising decision is made by trading off the net cost of equity against the net cost of debt. Lower liquid stocks have a higher inventory burden and cost, implying a higher net cost of equity financing. The debt financing strategy is more attractive; therefore, the leverage ratio is higher for lower liquid stocks (Nadarajah et al., 2018). Additionally, the pecking order theory claims that firms have a preference hierarchy from internal to debt to equity financing due to adverse selection costs resulting from information asymmetry. Stock liquidity decreases with information asymmetry, whereas debt financing increases with adverse selection and information asymmetry (Andres et al., 2014).

Theoretically, stock price, share volume, and stock return have a positive association with stock liquidity through inventory channels. Stoll (2000) explains that lower stock prices result in the evaporation of inventory value and higher inventory risk. Adverse stock price changes in the inventory lead to the risk of non-execution, which is referred to as the inventory risk of limited order traders (Rösch and Kaserer, 2013). The higher the possibility of fulfillment of limit orders, the lower the inventory and the lower the inventory risk (Hameed et al., 2010). Low historical stock returns are accompanied by low stock prices; therefore, there is a higher inventory risk. It is necessary to compensate for inventory risk with a wider bid–ask price spread or a higher expected return.

Institutional ownership and analyst coverage can affect stock liquidity through information asymmetry channels. Theoretically, the association with stock liquidity is mixed. The first stance claims a negative relationship between institutional ownership and stock liquidity. Institutional investors are perceived to be more experienced and better trained in processing information or even better informed. There is a large amount of asymmetric information between institutions and retail investors. Information asymmetry incurs a potential loss in trading against institutional investors. Uninformed retail investors require an adverse selection premium for their trade shares (Wang and Wei, 2021). The double trading cost reduces stock liquidity. The second stance argues that institutional participation can give rise to information transparency, improve corporate disclosure, and lower price uncertainty. Especially for China, accounting regulations have converged toward international standards in the last decade. Government disclosure reforms and anticorruption programs have been implemented in China. Insider trading activity is limited, and independent directors are available (Ding et al., 2017). Similarly, analysts can also promote information disclosure. Conversely, analysts have a greater incentive to follow stocks with more information asymmetry (Jiang et al., 2011). The relationship between analyst coverage and stock liquidity can be either negative or positive.

### Theoretical Effect of Systematic Factors on Stock Liquidity

Consumer price, M1, industry value-added, and market returns affect stock liquidity through the funding liquidity channel. Meanwhile, uncertainty affects stock liquidity through all the three channels.

Stock liquidity increases with market return through funding liquidity and wealth effect. Market return suffers an adverse reversal or decline with a drop in market-wide asset price. When asset price drops in the market, security wealth shrinks and market funding becomes constrained (Gârleanu and Pedersen, 2007). The spiral effect between funding liquidity and market liquidity decreases stock liquidity when funding is constrained (Amihud et al., 2013).

Consumer price, M1, and industry value-added, which are expected to be positive for stock liquidity, increase with expansionary monetary policy. Investors can easily meet the margin requirement to provide liquidity to the market under the expansionary monetary policy. Following this logic, the consumer price, M1, and industry value-added increase stock liquidity with monetary policy instruments. Few academic studies examine the impact of monetary policy on stock liquidity. Fernández-Amador et al. (2013) prove that expansionary monetary policy can strongly forecast 1-month ahead stock liquidity.

Stock liquidity declines with uncertainty through inventory risk, information symmetry, and funding constraint channels (Chordia et al., 2001; Amihud et al., 2013). Market uncertainty increases inventory risk. A higher expected return or larger price spread is required to compensate for inventory risk. Heightened market uncertainty increases information wedges between informed and uninformed investors. Unexpected productivity drop, excessive inflationary pressure, price reduction, and increasing real economy volatility can all induce fund outflows and deteriorate funding liquidity (Goyenko and Ukhov, 2009). Under the same theoretical mechanism as uncertainty, stock liquidity decreases with market volatility (Chung and Chuwonganant, 2018).

### Empirical Research in the Chinese Market

Information has a significant impact on Chinese stock liquidity. Gerace et al. (2015) find that the dissemination of indicative trade information during pre-open call auction sessions improves stock liquidity in a continuous trading session in China. Cheng et al. (2021) find that retail investor attention has a significantly positive short-term effect on future stock liquidity, and this effect will persist in the long-term future. Ding et al. (2017) study the effect of institutional ownership on stock liquidity from real and information friction perspectives. They find that foreign institutional participation leads to an increase in stock liquidity, but domestic institutional ownership significantly decreases the stock liquidity.

The impact of the regulatory reform on stock liquidity has been discussed based on the special features of the Chinese market. Ye et al. (2020) find that the impact of margin trading and short selling on stock liquidity reverses during market downturns. Li et al. (2018) use a natural experiment to show that the introduction of short selling significantly improves stock liquidity.

The literature discusses whether financial, economic, and accounting variations influence Chinese stock liquidity. Lee and Wong (2012) construct a financial liberalization index with three macroeconomic and three financial variables to establish that financial liberalization has a significant and positive impact on Chinese stock liquidity. Chu et al. (2015) indicate that Chinese listed firms with larger control–ownership divergences have poorer stock liquidity. Chen et al. (2013) find that average daily price and institutional proportion are the two most important determinants of Chinese stock liquidity. Ma et al. (2018) report that market return, GDP, and CPI significantly increase market liquidity.

## Data Sources, Sample Construction, and Variable Measurement

This section explains the data sources, sample selection standards, and variable measurements and adjustments. Table 1 shows the descriptive statistics for all variables. Correlations among all variables are less than 0.5 in the Table 2. Most of the independent variables even have very low pairwise correlation. Therefore, the coefficients in the following paper have less multicollinearity problem.

**Table 1:** Summary statistics.

	**N**	**Mean**	**Std. Dev**.	**Median**	**p10**	**p90**	**min**	**max**
logAmihud	451217	−3.309	1.343	−3.37	−4.959	−1.553	−6.461	0.318
logRoll impact	446947	−7.138	1.330	−7.181	−8.792	−5.416	−10.289	−3.612
logQspread	449980	−1.731	0.454	−1.741	−2.305	−1.129	−2.833	−0.631
logDepth	451217	20.86	1.398	20.903	19.057	22.618	17.151	24.11
IDIORET	451217	−0.007	0.024	−0.008	−0.032	0.02	−0.077	0.08
IDIORISK	451217	0.007	0.016	0.007	−0.008	0.025	−0.047	0.055
ADJ_TANG	451217	0.077	0.069	0.057	0.021	0.163	0.005	0.352
ADJ_LEV	451217	0.155	0.097	0.132	0.052	0.292	0.022	0.478
ADJ_ANACOV	451127	0.000	0.089	0.025	−0.093	0.069	−0.866	0.098
ADJ_FINST	451112	0.000	0.216	−0.003	−0.28	0.275	−0.57	10.517
ADJ_DINST	451217	−3.282	9.757	−5.874	−11.398	9.903	−14.207	67.862
ADJP_ASSET	451217	−1.622	1.500	−1.744	−2.322	−0.961	−2.709	58.38
ADJP_PRICE	451217	−0.252	22.470	−0.352	−32.716	29.299	−61.428	76.565
SYSRET	451031	−2.418	1.521	−2.64	−4.131	−0.5	−10.884	6.051
SYSRISK	451217	−0.369	0.698	−0.385	−1.234	0.499	−3.035	4.5
GRCP	451217	2.371	1.681	2.1	0.8	4.9	−1.8	8.7
GRM1	451217	11.388	7.735	9.26	3.3	22.1	0	38.96
IndVAG	451217	8.259	5.337	6.9	0	16.5	−1.1	23.2

**Table 2:** Pairwise correlations among independent variables.

**Variables**	**(1)**	**(2)**	**(3)**	**(4)**	**(5)**	**(6)**	**(7)**	**(8)**	**(9)**	**(10)**	**(11)**	**(12)**	**(13)**	**(14)**
(1) IDIORET	1.000													
(2) IDIORISK	0.003	1.000												
(3) ADJ_TANG	−0.035	−0.035	1.000											
(4) ADJ_LEV	−0.028	−0.006	0.467	1.000										
(5) ADJ_ANACOV	−0.002	0.001	0.005	−0.071	1.000									
(6) ADJ_FINST	−0.004	−0.005	−0.008	−0.055	0.105	1.000								
(7) ADJ_DINST	0.020	0.024	0.028	−0.095	−0.004	0.029	1.000							
(8) ADJP_ASSET	−0.004	0.000	−0.002	−0.043	0.015	0.031	0.118	1.000						
(9) ADJP_PRICE	0.017	−0.003	−0.060	−0.153	0.071	0.200	0.210	−0.001	1.000					
(10) SYSRET	0.004	−0.010	−0.075	−0.302	0.071	0.456	0.397	0.144	0.401	1.000				
(11) SYSRISK	0.032	0.040	0.055	0.172	−0.018	−0.244	0.438	0.069	0.017	−0.095	1.000			
(12) GRCP	−0.040	−0.012	−0.078	0.058	−0.001	0.000	0.045	−0.002	0.001	0.023	0.043	1.000		
(13) GRM1	−0.004	0.016	0.395	0.156	0.000	0.000	0.091	0.095	0.000	0.102	0.085	−0.022	1.000	
(14) IndVAG	−0.038	0.140	−0.029	−0.019	−0.001	0.000	0.064	0.144	−0.003	0.215	0.118	0.205	0.414	1.000

### Data Sources and Sample Construction

All the data are retrieved from the China Stock Market & Accounting Research (CSMAR) database. This study chooses accounting-related data from consolidated financial statements that reflect the complete operating information for the entire firm. To avoid value bias resulting from foreign exchange rate spot changes and spillover effects from other countries, A shares on the main board of the Shanghai and Shenzhen Stock Exchange markets, as well as ChiNext and SSE Star Market are selected.

Considering data availability and completeness, we collect data from January 2003 to September 2021. We only keep shares with a normal trading status and more than 10 trading days per month. The first-year trading samples after the IPO for every stock are deleted. The variables at the 1st and 99th percentiles are winsorized.

### Liquidity Measurement

Price impact, trading cost, trading speed, and trading quantity are the four stock liquidity dimensions. Price impact reflects the price reaction to the trading volume (Liu, 2006). Amihud's illiquidity ratio is the best liquidity proxy for capturing price impact in the stock market (Fong et al., 2017). It has been widely applied to measure stock liquidity in the Chinese stock market (Li et al., 2018; Ye et al., 2020). Roll's effective price spread is a traditional measure to capture the trading cost. Roll's price impact measure is based on Roll's effective price spread to show the impact of price on trading volume. Another popular measure of trading cost is the spread between bid and ask prices, which is a natural measure of illiquidity. The relative bid–ask spread is constructed with microstructure data, and it is a finer and better measure of illiquidity (Amihud, 2002). In addition to turnover, many traditional studies use bid and ask trading volumes to measure liquidity depth (Datar et al., 1998).

As the Chinese stock market is order-driven, we use trading volume to measure liquidity depth. This study also chooses Amihud's illiquidity ratio (Equation 1), Roll's price impact (Equation 2), and the quoted relative bid–ask spread (Equation 3) as proxies for stock liquidity. We use the logarithm of the four liquidity measures to test stock liquidity in China.

(1) $$\begin{array}{l} {Amihud}_{i,t}=\frac{1}{D_{i,t}}\overset{D_{i,t}}{\underset{d=1}{\sum }}\frac{\left|R_{i,td}\right|}{{VOLRMB}_{i,td}} \end{array}$$

where D_i,t_ is the number of valid trading days of stock *i* in month *t*. R_i,td_ is the daily return of stock *i* on day *d* of month *t* with cash dividends reinvested. VOLRMB_i,td_ is the daily trading volume value calculated in RMB currency for stock *i* on day *d* of month *t*. Amihud_i,t_ is the monthly Amihud's illiquidity ratio for stock *i*, which is the equally average daily Amihud's illiquidity ratio.

(2) $$\begin{array}{l} {Roll\text{\_}impactd}_{i,t}\\ =\{\begin{array}{ll} \frac{2\sqrt{-cov\left(\Delta P_{i,t},\Delta P_{i,t-1}\right)}}{{VOLRMB}_{i,t}} & ,when\;cov\left(\Delta P_{i,t},\Delta P_{i,t-1}\right)<0\\ 0 & ,when\;cov\left(\Delta P_{i,t},\Delta P_{i,t-1}\right)\geq 0 \end{array} \end{array}$$

where P_i,t_ is the daily return of stock *i* over month *t* and with cash dividends reinvested. ΔP_i,t_ is the first difference in the daily return. Roll_impact_i,t_ is the monthly impact of Roll's price on stock *i*.

(3) $$\begin{array}{l} {Qspread}_{i,t}=\frac{1}{D_{i,t}}\overset{D_{i,t}}{\underset{d=1}{\sum }}\frac{{Ask}_{i,td}-{Bid}_{i,td}}{M_{i,td}} \end{array}$$

where Ask_i,td_ is the daily selling price of stock *i* in month *t*. Bid_i,td_ is the daily buying price of stock *i* in month *t* and M_i,t_ is the midpoint price between the buying and selling prices of stock *i* in month *t*. The weight of the daily ask and bid prices is the trading amount in each transaction. Qspread_i,t_ is the monthly quoted relative price spread and is calculated using an equally average daily quoted relative price spread.

### Idiosyncratic Factors Measurement

Idiosyncratic return (IDIORET) is the residual part of individual stock returns in the capital asset pricing model (CAPM) (Equation 4). Daily residual stock returns are estimated according to the data of the latest 250 trading days based on the CAPM. Monthly idiosyncratic returns are the equally average daily residual stock returns in the month. Monthly idiosyncratic risk (IDIORISK) is measured using the variance of daily residual stock returns in the month.

(4) $$\begin{array}{l} r_{i,d}=r_{f,d}+\alpha_{i}\left(r_{m,d}-r_{f,d}\right)+\varepsilon_{i,d} \end{array}$$

where r_i,d_ is the daily return of individual stocks with cash dividend reinvestment, r_m,d_ is the daily market return with cash dividend reinvestment using the total market value-weighted average method, and r_i,f_ is the daily risk-free rate.

Tangibility (TANG) is a tangible asset ratio constructed by dividing total tangible assets by total assets. Previous results for the American and Australian markets show a positive relationship between the tangible asset ratio and stock illiquidity proxies (Nadarajah et al., 2018).

Leverage (LEV) is financial leverage, defined as total debt divided by total asset. Based on static trade-off and pecking order theories, the liquidity–leverage relation is expected to be negative through the channels of inventory cost and information asymmetry (Nadarajah et al., 2018).

Foreign institutional ownership (FINST) is the ratio of qualified foreign institutional holdings to the total A shares of a listed company. Domestic institutional ownership (DINST) is the percentage of shares held by domestic institutions, such as funds, security brokerages, insurance companies, social security funds, trust, finance companies, banks, and non-financial listed companies in China. Ding et al. (2017) find that foreign institutional ownership can enhance stock liquidity, whereas domestic institutional ownership significantly decreases stock liquidity in the Chinese stock market.

Tangibility, leverage, foreign institutional ownership, and domestic institutional ownership are quarterly data in the CSMAR. This study fills the same value every month in the same quarter. We adjust these variables with the market value to obtain the idiosyncratic part of these factors. ADJ_TANG, ADJ_LEV, ADJ_FINST, and ADJ_DINST refer to market-adjusted tangibility, leverage, foreign institutional ownership, and domestic ownership, respectively.

Analyst coverage (ANACOV) is measured by the number of analysts (teams) who have conducted a tracking analysis of a company in a year. One team is presented as 1, without counting the number of its members. The more the analyst (team) following, the more information disclosure. However, some argue that analysts have a greater incentive to follow stocks with greater information asymmetry (Jiang et al., 2011). Similarly, this study fills the same value every month of the same year. ADJ_ANACOV is the market-adjusted analyst coverage that presents the idiosyncratic part of this factor.

Asset (ASSET) is the total asset book value. Investors and analysts can easily pay attention to and scrutinize larger firms, which promotes the disclosure of firm fundamental information (Poon et al., 2013). Larger companies are expected to have higher liquidity levels. Price (PRICE) is the monthly closing price of individual stocks. Stoll (2000) explains that lower stock prices result in inventory value loss and higher inventory risk. A higher price spread is required to compensate for value loss and risk bearing. ADJP_ASSET and ADJP_PRICE represent asset and stock price changes relative to the market level, which is calculated using the logarithmic difference between individual variables and market levels.

### Systematic Factor Measurement

Systematic return (SYSRET) for an individual stock is the predictable part of the individual stock returns in the CAPM (Equation 4). Daily predictable stock returns are estimated according to the data of the latest 250 trading days based on the CAPM. A monthly systematic return is the equally average daily predictable stock return for the month. The monthly systematic risk (SYSRISK) is measured by the variance of daily predictable stock returns in the month.

The monthly growth rate of consumer price (GRCP) is the percentage change in the consumer price relative to the same month of the previous year. The monthly growth rate of M1 (GRM1) is the rolling 12-monthly growth rate of M1. Monthly industry value-added growth (IndVAG) is the year-to-year percentage change in industrial value-added for each month. The growth rates of consumer price and M1 are related to monetary policy. Fernández-Amador et al. (2013) suggest that expansionary monetary policy non-linearly increases aggregate stock market liquidity in the eurozone. The growth rates of consumer price and M1 are expected to be positive for stock liquidity.

## Contemporaneous Power Analysis of Stock Liquidity Determinants

The contemporaneous power of the determinants of stock liquidity is discussed by comparing idiosyncratic with systematic factors and analyzing the sensitivity variation of stock liquidity to determinants with economic policy uncertainty (EPU) change. The results show that idiosyncratic factors have more contemporaneous power to explain variations in stock liquidity than systematic factors. The sensitivities of stock liquidity to determinants are possible to increase, decrease or insignificantly change with EPU. These irregular results are related to variations in the investors' psychology.

### Contemporaneous Impact of Idiosyncratic and Systematic Factors on Stock Liquidity

To control for industry and time effects, this study uses a two-way fixed effects model to analyze panel data. This analysis in this subsection is based on baseline regression (5), wherein four liquidity proxies are tested to prove the robust impact of idiosyncratic and systematic factors on stock liquidity. X_i,t_ is the vector of idiosyncratic or systematic factors. This study includes 85 industries according to the 2012 China Securities Regulatory Commission classification.

(5) $$\begin{array}{r} Liquidity\;{Proxy}_{i,t}=\beta_{0}+\beta_{1}X_{i,t}+\chi_{k}\overset{84}{\underset{k=1}{\sum }}{Industry}_{k}\\ +\delta_{T}\overset{2020}{\underset{T=2003}{\sum }}{Year}_{T}+\varepsilon_{it} \end{array}$$

By comparing the *R*^2^ values in Table 3, we find that idiosyncratic factors are more powerful than systematic factors in explaining stock liquidity changes. This result implies that idiosyncratic determinants play a very important role in contemporaneous stock liquidity variation in China. The coefficients of the growth rate of consumer price, growth rate of M1, and industry value added growth are close to zero. These results further prove that the co-movement of stock liquidity caused by the macroeconomy is limited in the Chinese market. However, the systematic return and variance caused by the entire stock market have larger coefficients. This indicates that most stock liquidity co-movements are driven by stock market returns through individual return and variance channels. As theoretical expectation, the growth rate of consumer price and industry value added growth robustly increase stock liquidity over different liquidity measurements in the Chinese market.

**Table 3:** Contemporaneous impact of determinants on stock liquidity.

	**logAmihud**	**logRoll_impact**	**logQspread**	**logDepth**
**Panel A Idiosyncratic effect on stock liquidity**				
IDIORET	1.049 (22.81)^**^	1.232(25.80)^**^	0.558 (30.50)^**^	−1.856 (35.23)^**^
IDIORISK	0.312 (6.73)^**^	0.282 (6.16)^**^	−0.023 (1.05)	1.544 (30.55)^**^
ADJ_TANG	0.021 (0.33)	0.027 (0.43)	0.028 (1.09)	−0.096 (1.37)
ADJ_LEV	0.272 (5.94)^**^	0.197 (4.81)^**^	0.142 (7.27)^**^	−0.058 (1.62)
ADJ_ANACOV	−0.011 (16.06)^**^	−0.010 (15.20)^**^	−0.002 (8.09)^**^	0.010 (13.83)^**^
ADJ_FINST	−0.008 (2.05)^*^	−0.003 (0.83)	−0.008 (4.57)^**^	0.009 (2.46)^*^
ADJ_DINST	0.009 (31.80)^**^	0.009 (31.59)^**^	0.003 (25.51)^**^	−0.010 (31.02)^**^
ADJP_ASSET	−0.425 (41.48)^**^	−0.396 (40.64)^**^	−0.111 (28.43)^**^	0.360 (35.76)^**^
ADJP_PRICE	−0.510 (48.99)^**^	−0.548 (53.43)^**^	−0.368 (64.71)^**^	0.676 (58.61)^**^
Industry fix	Yes	Yes	Yes	Yes
Year fix	Yes	Yes	Yes	Yes
Constant	−1.165 (18.34)^**^	−5.137 (75.97)^**^	−1.081 (26.88)^**^	18.163 (211.56)^**^
N	451,016	446,747	449,779	451,016
R^2^	0.662	0.621	0.586	0.591
**Panel B Systematic effect on stock liquidity**				
SYSRET	−3.051 (58.62)^**^	−1.376 (24.53)^**^	−0.557 (28.57)^**^	2.582 (47.38)^**^
SYSRISK	−2.271 (49.58)^**^	−2.761 (61.20)^**^	−0.773 (34.28)^**^	4.749 (98.76)^**^
GRCP	0.013 (12.01)^**^	−0.003 (2.50)^*^	−0.004 (6.61)^**^	−0.006 (4.38)^**^
GRM1	−0.025 (83.53)^**^	−0.019 (61.45)^**^	−0.012 (86.19)^**^	0.012 (35.96)^**^
IndVAG	−0.012 (58.07)^**^	−0.012 (44.72)^**^	−0.004 (48.62)^**^	0.015 (58.08)^**^
Industry fix	Yes	Yes	Yes	Yes
Year fix	Yes	Yes	Yes	Yes
Constant	−1.631 (4.42)^**^	−5.716 (18.49)^**^	−1.026 (10.96)^**^	18.814 (64.31)^**^
N	451,217	446,947	449,980	451,217
R^2^	0.454	0.430	0.305	0.458

*This table shows the impact of 14 factors on stock liquidity. These factors are classified into two groups. Panel **A** shows how idiosyncratic factors change stock liquidity. Panel **B** shows how systematic factors change stock liquidity. The results in the panel A and panel B are based on regression (5) respectively. p-values are in parentheses*.

* *refers to p < 0.05*.

** *refers to p < 0.01. The asterisks in the following tables indicate the same significance levels*.

Except stock return and return variance, the impact of other idiosyncratic factors on stock liquidity are consistent with theoretical expectation. As previous research proves, the empirical results in Table 3 complement that only systematic component of stock return increases stock liquidity and only variance of idiosyncratic return decreases stock liquidity. On the other hand, variance of systematic return is found to significantly increase stock liquidity. Since the Chinese stock market uses an order-driven system, investors can easily enter and exit the market. Retail investors dominate the Chinese stock market. When they face idiosyncratic volatility, they have more fear and do not want to change their asset portfolio. Facing systematic volatility, retail investors may think that it is a common problem they encounter, and they tend to engage in speculative trading. This supplements a large amount of liquidity provision in the Chinese market due to the free entry–exit mechanism. The psychological response variation of retail investors makes it possible for systematic variance to increase stock liquidity. The second abnormal finding is that idiosyncratic return decreases contemporaneous stock liquidity. When not enough funding liquidity is promoted by idiosyncratic return, stock liquidity is possible to decrease with idiosyncratic return.

The impact of institutional ownership on stock liquidity is mainly determined by the balance between both information competition and disclosure effects. When the information disclosure effect is larger than the information competition effect, greater information symmetry makes institutional ownership stimulate stock liquidity. Institutional ownership usually increases the information disclosure effect through the promotion of international openness, corporate governance, and the capability to process information (Rhee and Wang, 2009). Foreign institutional ownership increases stock liquidity through larger information disclosure caused by international openness than information competition. The Chinese stock market is still dominated by retail investors, and domestic institutions want to hold more information to compete with retailers. The effect of information competition on the stock market increases with domestic institutional ownership. When the information disclosure effect is smaller than the information competition effect in China, information asymmetry increases and stock liquidity reduces with domestic institutional ownership. Our results show a negative impact of domestic institutional ownership and a positive impact of foreign institutional ownership on stock liquidity, which supports Ding et al.'s (2017) empirical result.

As much empirical research shows, our results also reveal that total assets and stock prices lead to an increase in stock liquidity. The negative liquidity–leverage relation in this research proves the previous theoretical analysis. The positive impact of analyst coverage on stock liquidity indicates that the former tends to increase information disclosure in China. Asset tangibility does not significantly decrease stock liquidity in China.

### Sensitivity of Stock Liquidity to Contemporaneous Variables Under Different EPU Levels

Contemporaneous impact analysis under high EPU is based on regression (6). D_t_ is 1 if EPU is larger than the average EPU. Otherwise, D_t_ is zero. Notably, EPU is the Chinese economic policy uncertainty index directly quoted from Baker et al. (2016). The average EPU for every month is the conditional mean value with a rolling window of 12 months prior. Z_i,t_ is the vector of all 14 determinants.

(6) $$\begin{array}{l} \;Liquidity\;{Proxy}_{i,t}\\ =\beta_{0}+\beta_{1}{D_{t}Z}_{i,t}+\beta_{2}Z_{i,t}+\chi_{k}\overset{84}{\underset{k=1}{\sum }}{Industry}_{k}\\ +\delta_{T}\overset{2020}{\underset{T=2003}{\sum }}{Year}_{T}+\varepsilon_{it} \end{array}$$

The sensitivities of stock liquidity to majority of contemporaneous variable are consistent for all four liquidity measures, when EPU is smaller than its average value (Table 4). The impact of idiosyncratic variance, tangibility, foreign institutional ownership, growth rate of M1, growth rate of consumer price and stock price on stock liquidity increases when EPU is above its mean level. Conversely, stock liquidity becomes less sensitive to leverage, asset, systematic variance and systematic return under higher EPU. The sensitivity of stock liquidity to other determinants does not change significantly or stably with EPU. These results imply that more attention is needed to be paid to idiosyncratic variance, idiosyncratic return, tangibility, foreign institutional ownership, growth rate of M1, growth rate of consumer price and stock price to control liquidity risk, while EPU increases. Because EPU promotes or irregularly changes the sensitity of stock liquidity to these variables, which induces more liquidity risk.

**Table 4:** The sensitivity of stock liquidity to contemporaneous determinants under different EPU levels.

	**logAmihud**	**logRoll_impact**	**logQspread**	**logDepth**
IDIORET	1.000 (17.54)^**^	0.906 (15.13)^**^	0.637 (27.67)^**^	−1.197 (19.03)^**^
IDIORET × D_t_	−0.332 (3.83)^**^	0.125 (1.43)	−0.200 (6.14)^**^	−0.519 (5.72)^**^
IDIORISK	−0.033 (0.53)	0.400 (6.64)^**^	−0.421 (14.82)^**^	0.327 (4.93)^**^
IDIORISK × D_t_	1.699 (34.06)^**^	1.527 (30.99)^**^	0.644 (32.65)^**^	−0.929 (17.45)^**^
ADJ_TANG	−0.062 (1.00)	−0.070 (1.14)	0.004 (0.17)	0.076 (1.18)
ADJ_TANG × D_t_	−0.042 (1.62)	−0.075 (2.91)^**^	0.017 (1.57)	0.093 (3.32)^**^
ADJ_LEV	0.467 (8.23)^**^	0.418 (7.87)^**^	0.168 (7.98)^**^	−0.380 (7.72)^**^
ADJ_LEV × D_t_	−0.126 (8.72)^**^	−0.112 (7.61)^**^	−0.013 (2.35)^*^	0.104 (6.60)^**^
ADJ_ANACOV	−0.012 (18.22)^**^	−0.012 (17.88)^**^	−0.003 (9.25)^**^	0.013 (18.76)^**^
ADJ_ANACOV × D_t_	0.001 (1.84)	0.000 (0.18)	0.000 (2.95)^**^	−0.001 (4.03)^**^
ADJ_FINST	0.000 (0.01)	0.004 (1.11)	−0.005 (3.02)^**^	−0.001 (0.28)
ADJ_FINST × D_t_	−0.008 (2.80)^**^	−0.008 (3.36)^**^	−0.003 (2.19)^*^	0.013 (3.06)^**^
ADJ_DINST	0.008 (30.80)^**^	0.008 (30.87)^**^	0.003 (23.87)^**^	−0.009 (32.27)^**^
ADJ_DINST × D_t_	0.000 (4.38)^**^	0.001 (5.06)^**^	0.000 (5.34)^**^	−0.000 (1.99)^*^
ADJP_ASSET	−0.485 (44.39)^**^	−0.470 (45.23)^**^	−0.115 (28.69)^**^	0.467 (44.63)^**^
ADJP_ASSET × D_t_	0.043 (19.75)^**^	0.043 (19.86)^**^	−0.002 (2.01)^*^	−0.040 (15.95)^**^
ADJP_PRICE	−0.397 (39.24)^**^	−0.415 (42.50)^**^	−0.347 (59.84)^**^	0.471 (45.94)^**^
ADJP_PRICE × D_t_	−0.028 (6.51)^**^	−0.016 (3.65)^**^	−0.011 (4.90)^**^	0.007 (1.49)
SYSRET	−5.684 (81.41)^**^	−2.121 (27.90)^**^	−1.065 (40.34)^**^	4.594 (58.61)^**^
SYSRET × D_t_	5.131 (54.14)^**^	1.481 (13.98)^**^	0.855 (24.05)^**^	−3.653 (36.10)^**^
SYSRISK	−2.533 (53.18)^**^	−2.913 (62.15)^**^	−0.417 (19.64)^**^	4.622 (88.82)^**^
SYSRISK × D_t_	0.515 (14.19)^**^	0.281 (7.90)^**^	0.121 (8.52)^**^	−0.302 (7.65)^**^
GRCP	−0.001 (1.35)	−0.015 (11.82)^**^	−0.004 (9.45)^**^	0.021 (14.11)^**^
GRCP × D_t_	0.020 (15.80)^**^	0.029 (20.07)^**^	−0.005 (8.62)^**^	−0.046 (29.46)^**^
GRM1	−0.021 (76.50)^**^	−0.016 (55.00)^**^	−0.010 (79.42)^**^	0.010 (31.90)^**^
GRM1 × D_t_	−0.009 (31.91)^**^	−0.006 (19.39)^**^	−0.004 (36.18)^**^	0.007 (19.94)^**^
IndVAG	−0.011 (47.61)^**^	−0.010 (34.60)^**^	−0.006 (57.18)^**^	0.016 (50.76)^**^
IndVAG × D_t_	0.001 (2.62)^**^	−0.001 (1.87)	0.003 (18.27)^**^	0.001 (2.69)^**^
Industry fix	Yes	Yes	Yes	Yes
Year fix	Yes	Yes	Yes	Yes
Constant	−0.402 (6.92)^**^	−4.483 (75.52)^**^	−0.744 (18.35)^**^	17.453 (256.73)^**^
N	451,016	446,747	449,779	451,016

*This table exhibits the different impact of factors on stock liquidity in the high EPU and low EPU. D_t_ is one if EPU is larger than the average EPU; otherwise, D_t_ is zero. The coefficients of factors indicate the sensitivities of stock liquidity to determinants in the low EPU. The coefficients of interaction terms indicate the difference between the impact of contemporaneous determinants on stock liquidity in the high EPU and the impact in the low EPU. The following empirical results are got from regression (6)*.

* *refers to p < 0.05.*

** *refers to p < 0.01*.

Results in the Table 4 also imply that tangibility, foreign institutional ownership, idiosyncratic variance and growth rate of consumer price mainly change contemporaneous stock liquidity under the high EPU.

## Predictive Power Analysis of Stock Liquidity Determinants

We discuss the predictive power of stock liquidity determinants in two ways. First, the 1-, 6-, and 12-month-ahead forecasts of stock liquidity are analyzed. Thereafter, the entire period is split into four subperiods, and a 1-month-ahead prediction is tested in every subperiod.

### Future Impact of the Determinants on Stock Liquidity

The analysis in this section is based on regression equation (7). F is the number of forward months. All the variables have the same meaning as in Equation (5).

(7) $$\begin{array}{l} \;{Liquidity\;Proxy}_{i,t+\;F}\\ =\beta_{0}+\beta_{1}X_{i,t}+\chi_{k}\overset{84}{\underset{k=1}{\sum }}{Industry}_{k}\\ +\delta_{T}\overset{2020}{\underset{T=2003}{\sum }}{Year}_{T}+\varepsilon_{it}\;\left(F=1,6,12\right) \end{array}$$

The *R*^2^ values in Tables 5, 6 show that the predictive power of idiosyncratic and systematic liquidity determinants generally decreases with the number of forward months. In every predicted month, idiosyncratic factors have more power to predict stock liquidity than systematic factors. All the five systematic factors significantly predict future stock liquidity. Much larger coefficients of systematic return and variance than other systematic factors imply that most of the predictive power is concentrated on systematic return and variance. The growth rates of M1 and consumer price are correlated with monetary policy. The results tell us that monetary policy has short-term effect on promoting stock liquidity. Inremental industry value added growth represents incremental real productivity and wealth, which can promote stock liquidity in the long term (e.g. 12 month in this research). Except idiosyncratic stock return and return variance, other idiosyncratic factors have consistent predictivity on stock liquidity over different predictive month and across different stock liquidity measurements, which perform as theory expect. Financial leverage, analyst coverage, institutional ownership, total asset and stock price are deduced to be stable drivers of stock liquidity in Chinese market.

**Table 5:** Future impact of variables on Amihud's illiquidity ratio and Roll's price impact.

	**logAmihud**			**logRoll_impact**		
	**F = 1**	**F = 6**	**F = 12**	**F = 1**	**F = 6**	**F = 12**
**Panel A Idiosyncratic determinants**						
IDIORET	−1.351 (30.33)^**^	−0.193 (4.19)^**^	−0.354 (7.67)^**^	−1.317 (27.70)^**^	0.070 (1.37)	−0.474 (9.53)^**^
IDIORISK	−0.307 (6.86)^**^	−2.183 (48.09)^**^	−1.737 (40.23)^**^	0.046 (1.00)	−1.276 (28.28)^**^	−1.286 (28.94)^**^
ADJ_TANG	0.066 (1.04)	0.145 (2.27)^*^	0.140 (2.06)^*^	0.061 (0.98)	0.124 (1.95)	0.103 (1.50)
ADJ_LEV	0.291 (6.59)^**^	0.347 (9.33)^**^	0.374 (9.47)^**^	0.229 (5.59)^**^	0.305 (8.22)^**^	0.326 (8.29)^**^
ADJ_ANACOV	−0.012 (17.48)^**^	−0.017 (23.74)^**^	−0.017 (21.81)^**^	−0.012 (17.73)^**^	−0.016 (24.03)^**^	−0.015 (20.85)^**^
ADJ_FINST	−0.016 (3.73)^**^	−0.005 (1.33)	−0.005 (1.42)	−0.015 (3.50)^**^	−0.002 (0.48)	−0.016 (3.19)^**^
ADJ_DINST	0.009 (31.06)^**^	0.005 (19.37)^**^	0.003 (11.82)^**^	0.008 (29.91)^**^	0.005 (18.57)^**^	0.003 (10.96)^**^
ADJP_ASSET	−0.408 (40.43)^**^	−0.372 (39.33)^**^	−0.350 (35.51)^**^	−0.383 (40.00)^**^	−0.352 (38.49)^**^	−0.326 (33.95)^**^
ADJP_PRICE	−0.494 (48.21)^**^	−0.339 (34.46)^**^	−0.227 (21.89)^**^	−0.501 (50.25)^**^	−0.341 (35.34)^**^	−0.238 (23.60)^**^
Industry fix	Yes	Yes	Yes	Yes	Yes	Yes
Year fix	Yes	Yes	Yes	Yes	Yes	Yes
Constant	−1.235 (19.17)^**^	−1.248 (20.34)^**^	−1.262 (18.18)^**^	−5.191 (74.07)^**^	−5.218 (83.66)^**^	−5.212 (70.14)^**^
N	440,215	409,401	384,341	436,293	405,571	380,702
R^2^	0.668	0.651	0.635	0.619	0.600	0.594
**Panel B Systematic determinants**						
SYSRET	−4.016 (83.10)^**^	−1.708 (34.43)^**^	1.915 (39.63)^**^	−3.947 (72.35)^**^	−1.112 (19.04)^**^	1.583 (26.67)^**^
SYSRISK	−2.301 (50.05)^**^	−1.774 (36.70)^**^	−1.407 (29.14)^**^	−2.362 (52.00)^**^	−1.499 (31.28)^**^	−1.271 (26.39)^**^
GRCP	0.003(2.60)^**^	0.103 (54.58)^**^	0.071 (47.72)^**^	−0.020 (15.06)^**^	0.090 (45.29)^**^	0.072 (47.52)^**^
GRM1	−0.012 (43.06)^**^	0.015 (48.47)^**^	0.005 (16.69)^**^	−0.000 (1.55)	0.011 (32.16)^**^	0.005 (14.11)^**^
IndVAG	0.002 (10.83)^**^	0.006 (26.34)^**^	−0.024 (92.49)^**^	0.003 (10.50)^**^	0.009 (34.08)^**^	−0.020 (67.30)^**^
Industry fix	Yes	Yes	Yes	Yes	Yes	Yes
Year fix	Yes	Yes	Yes	Yes	Yes	Yes
Constant	−2.163 (6.18)^**^	−3.065 (13.66)^**^	−2.470 (15.79)^**^	−6.350 (23.34)^**^	−6.946 (36.57)^**^	−6.398 (40.18)^**^
N	440,384	409,479	384,440	436,461	405,647	380,800
R^2^	0.454	0.435	0.419	0.422	0.405	0.401

*This table exhibits the predictive power of 14 determinants for 1-, 6- and 12-month ahead stock liquidity measured with Amihud's illiquidity ratio and Roll's price impact. The empirical analysis is based on regression (7), where F is the predicted step. Panel A shows the predictive power of idiosyncratic determinants for future stock liquidity and panel B shows the predictive power of systematic determinants for future stock liquidity*.

* *refers to p < 0.05*.

** *refers to p < 0.01*.

**Table 6:** Future impact of variables on quoted relative bid–ask spread and trading depth.

	**logQspread**			**logDepth**		
	**F = 1**	**F = 6**	**F = 12**	**F = 1**	**F = 6**	**F = 12**
**Panel A Idiosyncratic determinants**						
IDIORET	0.171 (9.89)^**^	0.116 (6.88)^**^	0.029 (1.61)	1.705 (31.64)^**^	−0.499 (31.64)^**^	0.716 (13.04)^**^
IDIORISK	−0.266 (13.04)^**^	−0.944 (46.67)^**^	−0.832 (41.04)^**^	1.162 (23.12)^**^	0.912 (17.36)^**^	0.467 (8.83)^**^
ADJ_TANG	0.041 (1.60)	0.061 (2.35)^*^	0.037 (1.25)	−0.124 (1.76)	−0.214 (2.88)^**^	−0.185 (2.30)^*^
ADJ_LEV	0.141 (7.62)^**^	0.125 (8.28)^**^	0.078 (4.64)^**^	−0.103 (2.78)^**^	−0.169 (4.14)^**^	−0.216 (4.90)^**^
ADJ_ANACOV	−0.002 (4.90)^**^	−0.004 (14.52)^**^	−0.005 (16.52)^**^	0.013 (17.41)^**^	0.019 (24.11)^**^	0.017 (19.67)^**^
ADJ_FINST	−0.010 (5.38)^**^	−0.006 (3.09)^**^	−0.005 (2.55)^*^	0.027 (5.02)^**^	0.001 (0.40)	0.024 (4.21)^**^
ADJ_DINST	0.003 (25.26)^**^	0.002 (15.84)^**^	0.001 (9.67)^**^	−0.009 (28.87)^**^	−0.006 (18.81)^**^	−0.004 (11.96)^**^
ADJP_ASSET	−0.107 (27.91)^**^	−0.084 (22.59)^**^	−0.060 (14.45)^**^	0.348 (35.11)^**^	0.306 (31.17)^**^	0.292 (27.53)^**^
ADJP_PRICE	−0.386 (68.24)^**^	−0.358 (69.77)^**^	−0.301 (60.04)^**^	0.586 (52.71)^**^	0.362 (33.94)^**^	0.232 (20.43)^**^
Industry fix	Yes	Yes	Yes	Yes	Yes	Yes
Year fix	Yes	Yes	Yes	Yes	Yes	Yes
Constant	−1.089 (28.01)^**^	−1.062 (26.68)^**^	−1.042 (22.36)^**^	18.150 (207.78)^**^	18.344 (241.17)^**^	18.437 (188.51)^**^
N	439,030	408,408	383,511	440,215	409,401	384,341
R^2^	0.603	0.609	0.568	0.588	0.550	0.537
**Panel B Systematic determinants**						
SYSRET	−1.097 (61.18)^**^	−0.550 (29.02)^**^	0.836 (44.74)^**^	4.768 (83.87)^**^	2.341 (37.09)^**^	−0.972 (14.70)^**^
SYSRISK	−0.878 (38.48)^**^	−0.847 (36.53)^**^	−0.718 (36.53)^**^	3.845 (79.50)^**^	1.883 (35.43)^**^	1.054 (19.39)^**^
GRCP	−0.005 (9.49)^**^	0.035 (52.92)^**^	0.032 (52.92)^**^	0.024 (16.30)^**^	−0.056 (26.73)^**^	−0.085 (51.45)^**^
GRM1	−0.008 (54.75)^**^	0.004 (29.13)^**^	0.001 (8.12)^**^	−0.008 (23.53)^**^	−0.019 (50.66)^**^	−0.003 (8.51)^**^
IndVAG	0.001 (11.36)^**^	0.003 (31.40)^**^	−0.005 (47.75)^**^	0.002 (6.86)^**^	−0.016 (55.31)^**^	0.030 (92.92)^**^
Industry fix	Yes	Yes	Yes	Yes	Yes	Yes
Year fix	Yes	Yes	Yes	Yes	Yes	Yes
Constant	−1.198 (13.47)^**^	−1.470 (20.16)^**^	−1.267 (18.32)^**^	19.385 (75.48)^**^	20.201(109.05)^**^	19.339 (112.46)^**^
N	439,199	408,486	383,610	440,384	409,479	384,440
R^2^	0.304	0.301	0.285	0.445	0.403	0.396

*This table exhibits the predictive power of idiosyncratic and systematic determinants for 1-, 6- and 12-month ahead stock liquidity measured with quoted relative bid–ask spread and trading depth. The empirical analysis also is based on regression (7), where F is the predicted step*.

* *refers to p < 0.05*.

** *refers to p < 0.01*.

Stock return and return variance have different predictive power from contemporaneous power for stock liquidity. In contrast to contemporaneous power of return for stock liquidity, stock return mostly increases future stock liquidity significantly. There is exception that idiosyncratic return still increases future relative bid-ask spread, which is similar to contemporaneous power of idiosyncratic return for stock liquidity. Facing larger systematic return, investors believe that the current funding liquidity is abundant and afraid of future funding liquidity evaporation. The believe induces more short-term trading and less long-term trading motivation. Therefore, systematic return is possible to robustly decreases 12-month forward stock liquidity. Return variance remarkably increases future stock liquidity over different predictive months and across different liquidity measurements. This indicates that short-term speculative psychology can drive long-term investor behavior. Current market status can change investor's expctation for the future stock market.

### Split Sample Analysis Over Time for Stock Liquidity Predictability

This research span is divided into four subperiod research groups. The first group is from January 2003 to December 2007 (T1). In the second subperiod (T2: 2008.01–2012.12), China also suffers from a negative impact of the American subprime financial crisis and the international debt crisis. The world experienced financial depression during the second subperiod. The third sub-period (T3: 2013.01–2017.12) covers the Chinese stock market crash period from June 2015 to September 2015. However, other countries entered the economic recovery stage in the third subperiod. The fourth period is from January 2018 to September 2021.

In Tables 7, 8, a 1-month forward prediction for stock liquidity is used to conduct the analysis in this subsection (Equation 7 on the condition that F is one). The growth rates of consumer price and M1, and industry value added growth have a minimal and unstable impact on 1-month forward stock liquidity over four subperiods. Except for idiosyncratic return and return variance, other idiosyncratic factors are consistent and significant to predict 1-month forward stock liquidity over four subperiods and across four liquidity measurements.

**Table 7:** One-month-forward prediction of Amihud's illiquidity ratio and Roll's price impact over four sub-periods.

	**logAmihud**				**logRoll_impact**			
	**T1**	**T2**	**T3**	**T4**	**T1**	**T2**	**T3**	**T4**
IDIORET	−1.367 (11.42)^**^	−1.676 (16.97)^**^	−1.435 (20.01)^**^	−1.476 (19.66)^**^	−1.383 (10.56)^**^	−1.689 (16.13)^**^	−1.499 (19.67)^**^	−1.358 (16.85)^**^
IDIORISK	0.754 (6.09)^**^	0.690 (7.15)^**^	0.581 (6.87)^**^	0.857 (9.59)^**^	1.103 (8.82)^**^	1.132 (11.38)^**^	0.953 (11.31)^**^	1.196 (13.32)^**^
ADJ_TANG	−0.272 (1.63)	−0.213 (2.02)^*^	−0.241 (2.53)^*^	−0.030 (0.24)	−0.251 (1.54)	−0.234 (2.22)^*^	−0.250 (2.74)^**^	−0.016 (0.12)
ADJ_LEV	0.745 (9.77)^**^	0.531 (7.06)^**^	0.385 (6.44)^**^	0.426 (3.63)^**^	0.697 (9.47)^**^	0.471 (6.46)^**^	0.328 (5.41)^**^	0.366 (3.46)^**^
ADJ_ANACOV	−0.010 (5.40)^**^	−0.013 (11.01)^**^	−0.011 (11.03)^**^	−0.014 (11.77)^**^	−0.010 (5.81)^**^	−0.014 (11.60)^**^	−0.011 (11.20)^**^	−0.014 (11.83)^**^
ADJ_FINST	−0.003 (0.39)	−0.022 (2.18)^*^	−0.019 (2.35)^*^	−0.001 (0.28)	−0.009 (1.01)	−0.028 (2.69)^**^	−0.018 (2.03)^*^	−0.003 (0.58)
ADJ_DINST	0.009 (14.67)^**^	0.008 (17.05)^**^	0.008 (16.96)^**^	0.008 (17.87)^**^	0.009 (14.20)^**^	0.007 (16.94)^**^	0.007 (15.97)^**^	0.008 (17.22)^**^
ADJP_ASSET	−0.407 (17.27)^**^	−0.478 (19.81)^**^	−0.520 (30.27)^**^	−0.399 (22.92)^**^	−0.382 (17.21)^**^	−0.452 (19.45)^**^	−0.491 (29.97)^**^	−0.388 (23.92)^**^
ADJP_PRICE	−0.564 (19.24)^**^	−0.398 (19.26)^**^	−0.368 (22.98)^**^	−0.459 (26.05)^**^	−0.572 (20.17)^**^	−0.400 (19.61)^**^	−0.364 (23.35)^**^	−0.454 (26.89)^**^
SYSRET	−4.284 (38.39)^**^	−3.292 (31.14)^**^	−3.612 (40.65)^**^	−4.222 (57.63)^**^	−4.445 (32.90)^**^	−3.142 (26.41)^**^	−3.468 (35.71)^**^	−4.124 (49.12)^**^
SYSRISK	−1.840 (16.14)^**^	−2.421 (27.29)^**^	−2.213 (35.93)^**^	−2.121 (27.21)^**^	−1.924 (16.66)^**^	−2.620 (29.05)^**^	−2.299 (37.72)^**^	−2.253 (29.33)^**^
GRCP	0.011 (3.87)^**^	−0.012 (4.80)^**^	−0.003 (1.38)	0.009 (4.94)^**^	−0.003 (0.90)	−0.031 (10.63)^**^	−0.030 (12.44)^**^	−0.008 (4.06)^**^
GRM1	−0.015 (22.91)^**^	−0.010 (18.32)^**^	−0.013 (24.77)^**^	−0.012 (28.92)^**^	−0.004 (5.50)^**^	0.002 (3.53)^**^	0.001 (1.68)	−0.002 (4.30)^**^
IndVAG	0.007 (12.25)^**^	0.004 (7.35)^**^	−0.002 (4.45)^**^	0.004 (10.71)^**^	0.004 (5.94)^**^	0.003 (5.72)^**^	0.002 (4.68)^**^	0.004 (8.60)^**^
Industry fix	Yes	Yes	Yes	Yes	Yes	Yes	Yes	Yes
Year fix	Yes	Yes	Yes	Yes	Yes	Yes	Yes	Yes
Constant	−1.572 (14.43)^**^	−0.294 (1.63)	−0.709 (6.21)^**^	−1.249 (14.85)^**^	−5.692 (55.46)^**^	−4.309 (24.14)^**^	−5.028 (36.20)^**^	−5.324 (63.41)^**^
N	64,247	91,225	137,144	147,599	63,673	90,454	135,921	146,245

*Idiosyncratic determinants have more stable and consistent influence on 1-month forward stock liquidity than systematic determinants over four subperiods, when the stock liquidity is measured with Amihud's illiquidity ratio and Roll's price impact. The first subperiod (T1) is from January 2003 to December 2007. The second subperiod (T2) is from January 2008 to December 2012. The third subperiod (T3) is from January 2013 to December 2017. The fourth subperiod (T4) is from January 2018 to September 2021*.

* *refers to p < 0.05*.

** *refers to p < 0.01*.

**Table 8:** One-month-forward prediction of quoted relative bid–ask price spread and trading depth over four sub-periods.

	**logQspread**				**logDepth**			
	**T1**	**T2**	**T3**	**T4**	**T1**	**T2**	**T3**	**T4**
IDIORET	0.190 (4.28)^**^	0.073 (1.84)	0.193 (6.53)^**^	0.159 (5.51)^**^	1.762 (12.59)^**^	2.050 (17.82)^**^	2.085 (24.46)^**^	1.990 (22.14)^**^
IDIORISK	−0.072 (1.34)	−0.188 (3.76)^**^	−0.110 (2.62)^**^	−0.055 (1.33)	−0.432 (3.16)^**^	−0.490 (4.77)^**^	−0.308 (3.40)^**^	−0.511 (5.29)^**^
ADJ_TANG	0.016 (0.30)	−0.108 (2.47)^*^	−0.019 (0.48)	0.062 (1.01)	0.241 (1.35)	0.283 (2.50)^*^	0.231 (2.40)^*^	0.063 (0.46)
ADJ_LEV	0.206 (7.57)^**^	0.228 (8.02)^**^	0.136 (5.33)^**^	0.187 (3.76)^**^	−0.675 (9.23)^**^	−0.409 (5.38)^**^	−0.262 (4.25)^**^	−0.357 (3.65)^**^
ADJ_ANACOV	0.000 (0.23)	−0.003 (5.15)^**^	−0.003 (5.84)^**^	−0.003 (4.99)^**^	0.012 (6.44)^**^	0.015 (11.97)^**^	0.012 (11.28)^**^	0.015 (12.60)^**^
ADJ_FINST	−0.007 (1.82)	−0.016 (2.76)^**^	−0.013 (3.47)^**^	−0.003 (1.24)	0.014 (1.44)	0.044 (3.20)^**^	0.040 (3.07)^**^	0.009 (1.64)
ADJ_DINST	0.002 (9.80)^**^	0.003 (13.57)^**^	0.003 (13.15)^**^	0.003 (13.83)^**^	−0.010 (13.95)^**^	−0.008 (16.22)^**^	−0.007 (16.16)^**^	−0.009 (17.68)^**^
ADJP_ASSET	−0.092 (13.60)^**^	−0.132 (16.29)^**^	−0.133 (18.18)^**^	−0.102 (13.73)^**^	0.380 (16.71)^**^	0.447 (19.52)^**^	0.479 (28.55)^**^	0.380 (23.45)^**^
ADJP_PRICE	−0.470 (27.74)^**^	−0.359 (30.65)^**^	−0.333 (36.22)^**^	−0.386 (36.71)^**^	0.580 (20.31)^**^	0.419 (19.36)^**^	0.394 (23.84)^**^	0.497 (27.74)^**^
SYSRET	−1.266 (34.20)^**^	−1.066 (29.27)^**^	−1.012 (30.67)^**^	−1.154 (43.88)^**^	5.464 (42.53)^**^	3.762 (30.18)^**^	4.183 (39.93)^**^	5.207 (60.02)^**^
SYSRISK	−0.319 (6.55)^**^	−0.463 (11.42)^**^	−0.391 (12.22)^**^	−0.410 (11.94)^**^	3.247 (26.68)^**^	3.943 (40.71)^**^	3.590 (54.18)^**^	3.561 (41.77)^**^
GRCP	−0.009 (7.62)^**^	−0.008 (6.88)^**^	−0.006 (6.84)^**^	−0.006 (7.48)^**^	0.011 (3.29)^**^	0.038 (11.28)^**^	0.034 (12.53)^**^	0.019 (7.93)^**^
GRM1	−0.010 (33.97)^**^	−0.007 (22.93)^**^	−0.008 (32.03)^**^	−0.008 (39.37)^**^	−0.003 (3.45)^**^	−0.010 (15.42)^**^	−0.010 (14.80)^**^	−0.007 (13.54)^**^
IndVAG	0.001 (5.94)^**^	0.001 (4.92)^**^	−0.002 (9.24)^**^	−0.000 (0.16)	0.002 (2.60)^**^	0.002 (3.87)^**^	0.003 (5.80)^**^	0.001 (3.22)^**^
Industry fix	Yes	Yes	Yes	Yes	Yes	Yes	Yes	Yes
Year fix	Yes	Yes	Yes	Yes	Yes	Yes	Yes	Yes
Constant	−1.334 (38.32)^**^	−0.876 (9.33)^**^	−0.914 (18.75)^**^	−0.928 (15.59)^**^	18.837 (173.84)^**^	17.256 (75.59)^**^	18.053 (109.87)^**^	18.310 (215.36)^**^
N	64,238	91,150	136,057	147,585	64,247	91,225	137,144	147,599

*This table shows the 1-month forward prediction of determinants for the stock liquidity measured with quoted relative bid-ask price spread and trading depth. T1 to T4 are four subperiods the same with ones in the Table 7*.

* *refers to p < 0.05*.

** *refers to p < 0.01*.

Stock return increases all 1-month forward stock liquidity at any subperiod except predictive power of idiosyncratic return to relative bid-ask spread. Systematic variance robustly increases all kinds of 1-month forward stock liquidity at any subperiod, but idiosyncratic variance significantly decreases 1-month forward stock liquidity measured with Amihud's ratio, Roll's price impact and trading depth.

Generally speaking, stock return and systematic variance can robustly increase short-term future stock liquidity at any subperiods, but idiosyncratic variance decreases short-term future stock liquidity. Compared with macroeconomic factors, idiosyncratic factors related with corporate attributes have stronger and more stable short-term prediction for the stock liquidity.

## Conclusion

Return and volatility are two popular determinants of stock liquidity. Previous research focuses on how stock return and return volatility affect stock liquidity. They find a positive relationship between stock return and stock liquidity and a negative relationship between return volatility and stock liquidity. This study contributes to the literature by splitting individual return into predictable part driven by market return and idiosyncratic part. Similarly, return variance also is splite into systematic part and idiosyncratic part. The results support the notion that return variance and stock return have strong predictive and contemporaneous power for stock liquidity. This study further finds that idiosyncratic return abnormally decreases contemporaneous stock liquidity and systematic return also can decrease 1-year forward stock liquidity. Except for negative relation between idiosyncratic variance and contemporaneous stock liquidity, stock liquidity increases with return variance. These abnormal phenomenon might be caused by Chinese market mechanism and investors' trading psychology.

The literature usually focuses on the contemporaneous relationship between a single factor and stock liquidity. This study contributes to the literature by focusing on two groups of factors: systematic and idiosyncratic determinants. Through using four liquidity proxies, different numbers of month forward prediction and split sample analysis, systematic determinants are robustly found to have less contemporaneous and predictive power for stock liquidity than idiosyncratic determinants. Monetary policies have minimal and unstable influence on Chinese stock liquidity. Financial leverage, analyst coverage, institutional ownership, total asset and stock price robustly determine stock liquidity in Chinese market. These empirical results are helpful to further study effective determinants of stock liquidity in China.

Research has been conducted on how EPU affects stock liquidity. This study focuses on the impact of EPU on the sensitivity of stock liquidity to contemporaneous determinants. We find that EPU can change sensitivity of stock liquidity to determinants but the variation is irregular. Under the high EPU, we need especially focus on the factors which can make stock liquidity more sensitive.

## Data Availability Statement

The original contributions presented in the study are included in the article/supplementary materials, further inquiries can be directed to the corresponding author/s.

## Author Contributions

All authors listed have made a substantial, direct and intellectual contribution to the work, and approved it for publication.

## Funding

LX is a lecturer in the College of Science and Technology Ningbo University and a Phd student in the University of Nottingham Ningbo China. LX got funding project (G21-3-ZX03) from the third batch of Ningbo Academy of Social Sciences in 2021: the determinants and prevention of financial market liquidity risk in Ningbo.

## Conflict of Interest

The authors declare that the research was conducted in the absence of any commercial or financial relationships that could be construed as a potential conflict of interest.

## Publisher's Note

All claims expressed in this article are solely those of the authors and do not necessarily represent those of their affiliated organizations, or those of the publisher, the editors and the reviewers. Any product that may be evaluated in this article, or claim that may be made by its manufacturer, is not guaranteed or endorsed by the publisher.
